# Case report: A contralateral superficial cervical axial pattern flap in a large head and neck skin defect including a unilateral auricle

**DOI:** 10.3389/fvets.2025.1594631

**Published:** 2025-08-12

**Authors:** Kim Gwan-Yong, Kwak Ho-Hyun, Kim Jun-Hyung, Woo Heung-Myong

**Affiliations:** ^1^Department of Veterinary Surgery, College of Veterinary Medicine, Institute of Veterinary Science, Kangwon National University, Chuncheon, Republic of Korea; ^2^Department of Companion Animal Industry, College of Natural and Life Sciences, Daegu University, Gyeongsan, Republic of Korea

**Keywords:** dog, sensory organ, large head and neck skin defect, sugar dressing, contralateral axial pattern flap, Penrose drain pexy

## Abstract

A 3-year-old Shiba Inu was presented to the hospital with a large skin defect (15 cm × 25 cm, oval shape) on the head and neck, involving the left auricle, caused by a traffic accident. Necrotic tissue caused by inflammation was observed in the area of the skin defect. To address this condition, a sugar dressing was applied for 17 days to promote granulation tissue formation before performing a superficial cervical axial pattern flap. The flap was rotated by 120°, ensuring that the right superficial cervical artery remained undisturbed. The distal aspect of the flap was temporarily fixed to the forehead defect using a towel clamp before suturing. A 1 cm-long Penrose drain was pexied parallel to the vascular direction to reduce dead space. The grafted flap was adhered successfully without any significant complications. This case describes the successful treatment of a large head and neck skin defect, involving a sensory organ, using a contralateral axial pattern flap and Penrose drain pexy. This approach reduces the complications of axial-pattern flaps related to vascular twisting. Therefore, the use of contralateral superficial cervical axial pattern flap and Penrose drain pexy may be an effective solution for large head and neck skin defects, including auricular defects.

## 1 Introduction

Large skin defects in the head and neck regions of dogs commonly occur due to traffic accidents (1). The treatment of large skin defects in this region is challenging because they involve various composite tissues, making it difficult to reconstruct the individually severe different functions of the damaged tissue (2). Additionally, the soft tissue in this area has more limited skin tension than in other parts of the body, further complicating reconstruction (3). To reconstruct large skin defects in the head and neck, axial pattern flaps, such as the superficial temporal, superficial cervical, and caudal auricular axial pattern flaps, are relevant (3). Subdermal plexus flaps have been used to reconstruct periorbital skin defects (4). A caudal auricular axial-pattern flap has been used for auricular reconstruction (5). In addition, superficial cervical axial pattern flaps have been used for defects in the face, ear, neck, shoulder, and axillary regions (6). Although a case of the treatment of right axillary skin defects in cats using a superficial cervical axial pattern flap has been reported (7). there are no documented cases of its use for head and neck defects in dogs.

In the presented case, a superficial cervical axial pattern flap was selected because the defect involved areas where typically superficial temporal and caudal auricular axial pattern flaps would have been used. Furthermore, during a surgery involving a unilateral auricular defect, using a flap from the same side would have covered the ear canal and elevating and rotating the flap would have increased the risk of vascular embarrassment. Therefore, a contralateral axial-pattern flap was selected. The outcomes and complications of axial-pattern flaps include seromas, flap necrosis, and flap dehiscence (8). To reduce these complications, preservation of the blood supply and ensuring proper drainage and infection control have been reported (9); however, complications continue to occur. Surgical drains are recommended for managing dead spaces (10). In this case, the method used for auricular hematoma treatment was adapted to prevent hematoma formation. Instead of using buttons (11) and dental rolls (12) as supports to maintain equal compression, a 1-cm long Penrose drain was used and sutured to the fascia to prevent the formation of dead space.

The purpose of this report is to describe a case in which a contralateral axial pattern flap and Penrose drain pexy were used to reduce complications in the reconstruction of a large head and neck skin defect, including a unilateral auricular injury.

## 2 Case description

**Figure 1:**
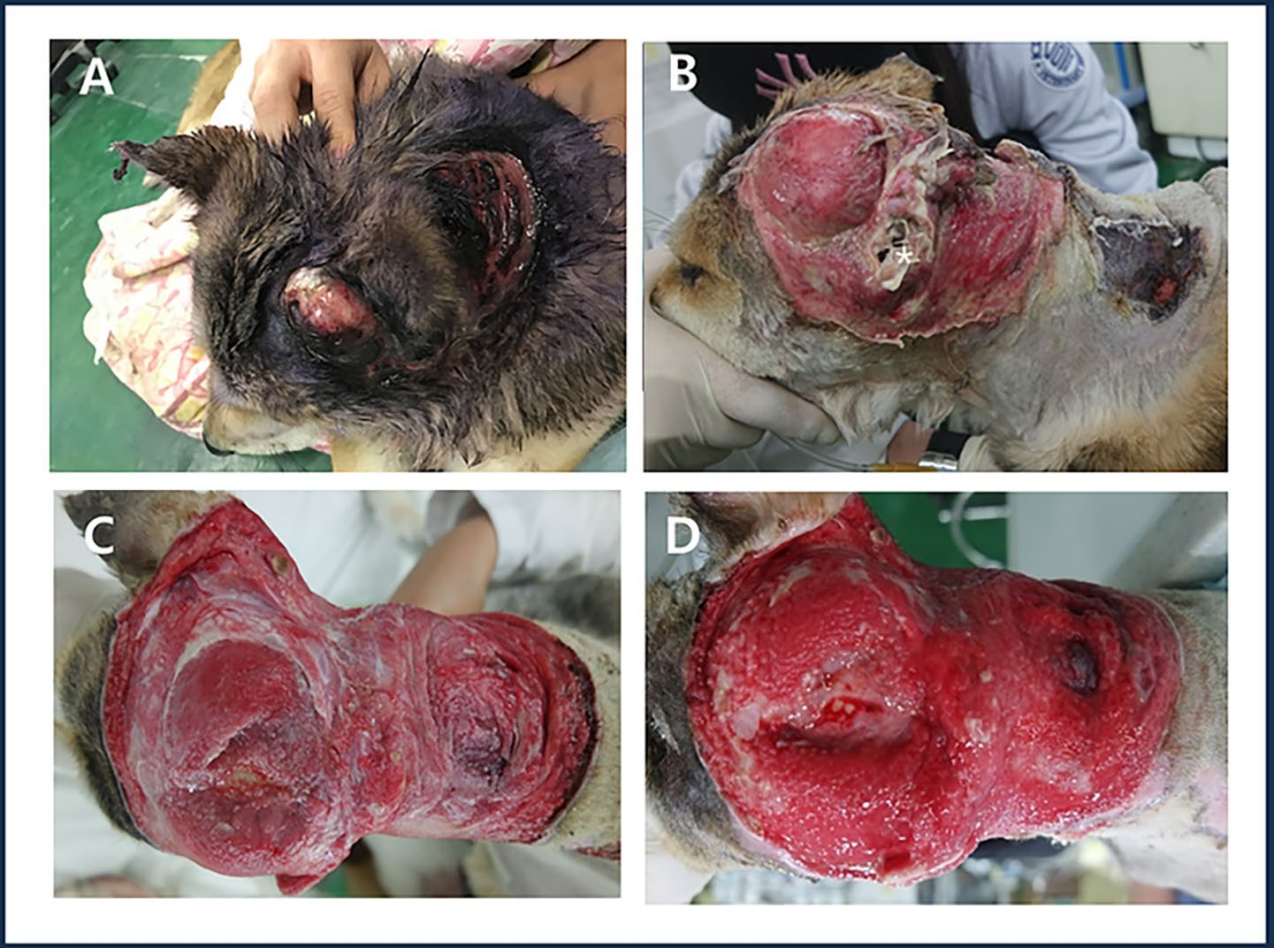
Large open wound in the head and neck with damage to the left auricle. **(A)** Patient’s appearance on the day of admission. **(B)** Wound condition after debridement of contaminants and necrotic tissue on the day of admission, showing the remaining external auditory canal (white asterisk). **(C)** Granulation process observed on day 7 after admission following necrotic tissue removal. **(D)** Formation of a healthy, well-vascularized granulation tissue layer observed on day 17 after admission.

A 3-year-old female Shiba Inu was presented with a severe large skin defect (15 cm × 25 cm, oval shape) on the head and neck, including the left pinna, caused by a traffic accident (Figure 1A). The dog went missing and was found on the roadside 7 days later. It was rescued and transferred from an animal shelter to the Kangwon National University Veterinary Teaching Hospital. On the day of admission, a physical examination revealed loss of the left pinna and necrotic tissue in the head and neck region due to inflammation (Figure 1B).

On the day of admission, necrotic tissue and surrounding skin were excised to prevent further contamination. The wound was then thoroughly irrigated with a sterile 0.9% saline solution using a 60-mL syringe and an 18-gauge needle under appropriate pressure. After irrigation, the surrounding area was dried using a sterile gauze, and a thick layer of sugar (at least 1–2 cm thick) was applied over the entire wound. A large amount of sterile absorbent gauze was used to secure the first layer with cotton bandages, followed by elastic bandages for additional fixation. Bandage was changed once or twice daily depending on the amount of exudate produced. The wound was initially irrigated with 1 L of saline and subsequently with 500–1,000 mL of saline during each bandage change. The sugar dressing and bandaging technique was continued for 17 days until the damaged tissue was covered and all necrotic tissue was removed. During this 17-day period, amoxicillin-clavulanate (15 mg/kg, intravenously [IV], every 12 h), metronidazole (10 mg/kg, IV, every 12 h), marbofloxacin (2 mg/kg, subcutaneously [SC], every 24 h), meloxicam (0.1 mg/kg, SC, every 24 h), and tramadol (3 mg/kg, IV, every 12 h) were administered.

Seventeen days after admission, the skin in the head and neck region appeared bright red with significantly reduced exudate and a well-formed healthy granulation tissue layer was observed (Figure 1D).

The dog was premedicated with butorphanol (0.2 mg/kg, IV) and midazolam (0.2 mg/kg, IV). Induction was performed with propofol (6 mg/kg, IV), and anesthesia was maintained with isoflurane in oxygen. Cefazolin (22 mg/kg, IV) was administered at induction.

**Figure 2:**
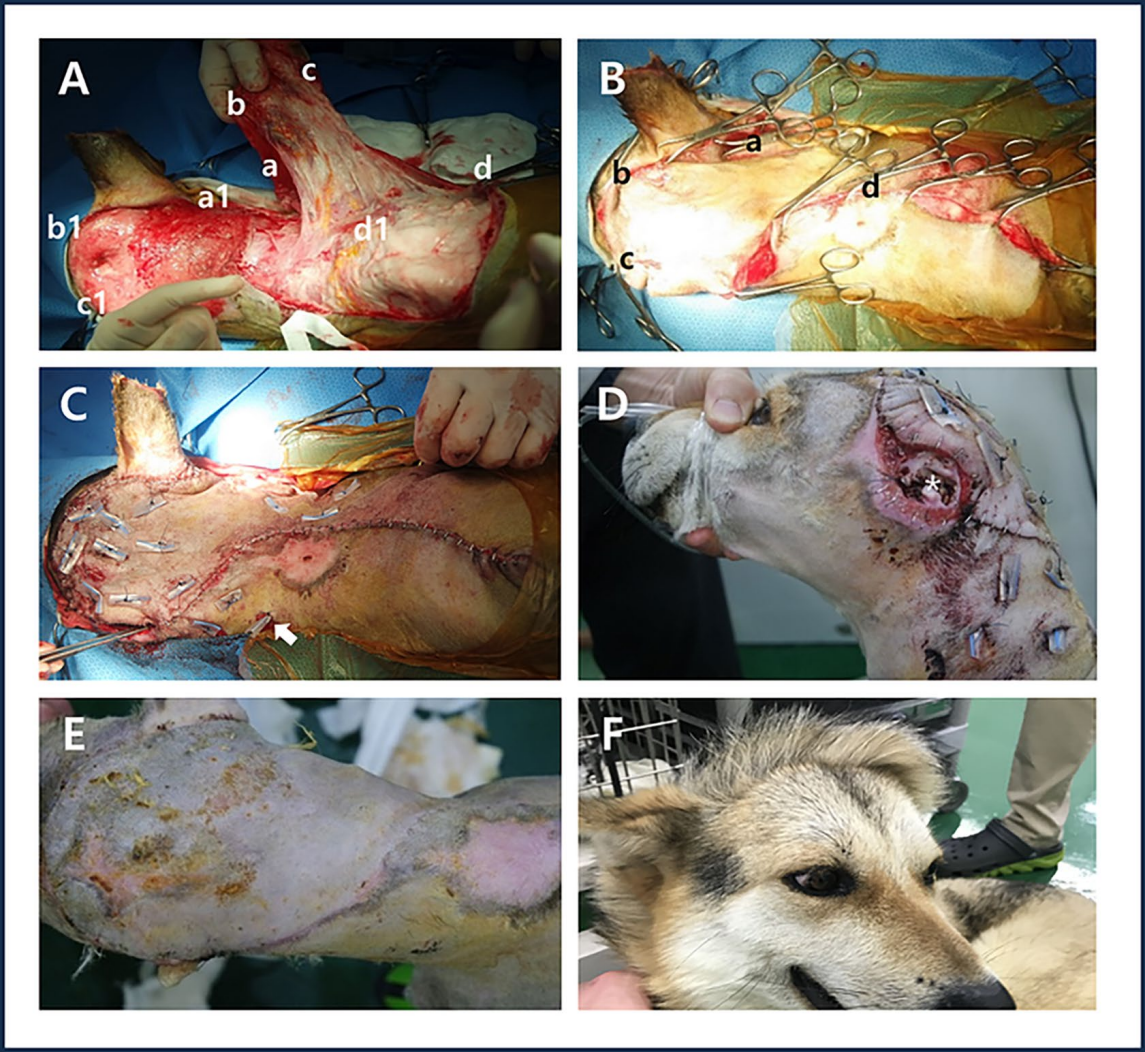
Surgery using a contralateral superficial cervical axial pattern flap and Penrose drain pexy. **(A)** In the illustrations of the surgical process, the midpoint **(a)** of the dorsal flap length was fixed to the caudal side of the right ear **(a1)**. Endpoint **(b)** of the remaining dorsal flap length was temporarily secured using a towel clamp at the starting point **(b1)** of the head defect. The endpoint **(c)** of the ventral flap length was fixed to the cranial side of the left ear **(c1)**, while the starting point **(d)** of the ventral flap length was secured to the cranial border of the right shoulder **(d1)**. **(B)** Temporary fixation using a towel clamp, the midpoint of the dorsal flap length **(a)** Endpoint of the remaining dorsal flap length **(b)** the endpoint of the ventral flap length **(c)** the starting point of the ventral flap length **(d)** after the flap transplantation, any remaining empty space was addressed by bluntly separating the residual cervical skin and temporarily securing it using a towel clamp. **(C)** Placement of the Penrose drain (white arrow) and simple suturing after applying Penrose drain pexy. **(D)** Appearance of the remaining ear canal (white asterisk) on postoperative day 7. **(E)** Appearance of the grafted area on postoperative day 14 after removal of sutures and the Penrose drain. **(F)** Observation of hair direction at the graft site 10 months postoperatively.

During the grafting process, the flap was rotated 120° and the midpoint of the dorsal flap length was fixed to the caudal side of the right ear. Endpoint of the remaining dorsal flap length was temporarily secured using a towel clamp at the starting point of the head defect. The endpoint of the ventral flap length was fixed to the cranial side of the left ear, while the starting point of the ventral flap length was secured to the cranial border of the right shoulder. After the flap transplantation, any remaining empty space was addressed by bluntly separating the residual cervical skin and temporarily securing it using a towel clamp (Figures 2A,B). A Penrose drain was inserted under the flap, extending from the right ear to the left shoulder. Rotation of the peninsula flap, the distal aspect of the flap, and the skin of the neck that remained after blunt dissection were pexied with a Penrose drain. A 1 cm-long Penrose drain pexy was used parallel to the vascular direction. The vascular bed of the flap was preserved as much as possible to prevent vascular damage, except at the base of the flap where the blood vessels were not clearly identifiable. The towel clamps were individually removed while performing simple suturing (Figure 2C).

The damaged portion of the ear canal under the left ear cartilage was sutured to the underlying skin using staples. Postoperatively, the patient was administered amoxicillin-clavulanate (15 mg/kg, IV, every 12 h), metronidazole (10 mg/kg, IV, every 12 h), meloxicam (0.1 mg/kg, SC, every 24 h), and tramadol (3 mg/kg, IV, every 12 h) for 14 days.

Seven days after surgery, no redness, odor, or exudate was observed in the damaged area of the left ear or the distal flap site, and the skin appeared normal (Figure 2D). Fourteen days after surgery, no dehiscence or inflammation was observed at the flap graft site; therefore, the sutures and Penrose drain were removed (Figure 2E). Ten months after surgery, the direction of hair growth in the grafted area was opposite to the original hair direction of the head and neck (Figure 2F).

## 3 Discussion

This case report describes the first documented use of a contralateral superficial cervical axial pattern flap and Penrose drain pexy in canines with large skin defects in the head and neck region, including damage to the auricle, a sensory organ, caused by a traffic accident. This surgical approach was implemented to enhance the survival rate of the axial-pattern flap while reducing complications.

In cases of head and neck defects involving sensory organ damage, preoperative evaluations are necessary to assess sensory impairments. In modern veterinary medicine, brainstem auditory evoked response testing is used to evaluate hearing impairments (13). In human medicine, auricular reconstruction using costal cartilage and silicone prostheses has been reported (14). The pinna acts as a sound funnel (15), and the difference between dogs and humans is the proportion of the external auditory meatus covered by cartilage or temporal bone, which accounts for approximately 30% in persons and up to 98% in dogs (16). Additionally, dogs have a cartilage at the caudal base of the pinna, which serves as an attachment point for several muscles (17).

Reconstruction of large skin defects in the head and neck, including sensory organs, should be accompanied by surgical treatment of the affected organs. Reconstruction using trephine and silicone stents with a superficial temporal tube flap has been performed to reduce defects and restore function in the dorsal nasal and rostral regions (2). Additionally, reconstruction using a superficial temporal axial pattern flap and nictitans with a silicone tube in the nasolacrimal duct has been documented to reduce defects in the medial eyelids and canthus while preserving corneal function (18).

In the present case, although the auricle was absent, the cartilage surrounding the ear canal remained intact. Therefore, rather than reconstructing the auricle, a contralateral axial-pattern flap was applied to the basal cartilage of the left pinna.

When the patient was first brought to the clinic, the wound was in the late inflammatory phase, more than 5 days post-trauma (19), and showed severe skin necrosis due to trauma (20). Granulation tissue is not required for axial-pattern flaps; however, the recipient bed should be free of gross contamination (19). If there is sufficient vasculature to accommodate a skin graft, the healing process can be significantly accelerated (21). Sugar dressings are used because of their high osmolality, improved debridement, and aid in the healing process by regenerating tissue (22). Both honey and sugar dressings have been reported to promote wound healing (23). The cotton bandages ensured that the sugar remained in place and covered the entire wound, with the first layer being sterile and highly absorbent (22). Sugar bandages must be changed frequently because of the exudative nature of the wounds and the quickly saturated sugar layers (22). A general rule of thumb to follow is when white, dry, granulated sugar is still present, bandage changes can be less frequent (22). In this case, preparing a recipient wound bed with abundant vessels through appropriate treatment with sugar dressings and cotton bandages, including the removal of contaminants and necrotic tissue, is critical for successful graft acceptance.

The cranial border of the caudal auricular axial pattern flap extends to the palpable depression between the lateral aspect of the wing of the atlas and the vertical ear canal (24). Because damage to the ear was included in the caudal auricular axial pattern flap, a superficial cervical axial pattern flap was chosen instead.

Choosing whether to apply the same- or opposite-side flap is crucial when dealing with large skin defects, including unilateral ear damage. If a same-side flap is used to cover the left ear defect, it covers the ear canal and leads to circulatory problems. When the flap is transposed at 180°, the risk of twisting the direct cutaneous artery and vein increases, further increasing the risk of vascular embarrassment (8). Furthermore, if a hole is created in the flap to expose the ear canal, the risk of losing the entire flap increases. However, by suturing the flap from the opposite side to the cartilage at the base of the pinna over the damaged ear canal, the area of the flap tissue can be reduced, thereby avoiding exposure of the ear canal and minimizing the risk to the flap.

Although axial pattern flaps carry their own blood supply, they depend on the continuation of adequate circulation (25). In veterinary literature, length-to-width ratios from 1:1 to 3:1 have been recommended to prevent complications (26). Reasons for avoiding bandages include the potential for vascular compromise of the flap if they are too tight (5). Drains prevent the buildup of subcutaneous fluid under the flap, which can lead to surgical failure (19). An axial pattern flap can be created as a peninsula or an island, with the peninsula flap having the drawback of resulting in a “dog ear” at its point of rotation (19). The island flap is more mobile than the peninsular flap but has limited practicality (6). Evaluations of the closure time, cosmetic appearance, and healing time of suturing techniques, skin staples, and surgical glue for auricular hematoma in dogs have been reported (27). Dental rolls can be secured with suture to the anterior and posterior site of the drained hematoma (12). Some authors recommend that suturation should involve hard materials such as buttons (28). The suture pexy of the pinna structure was placed at separate points and applied parallel to the major vessels (29). In a study on color-flow Doppler ultrasound of direct cutaneous arteries used for axial pattern flaps in dogs, the superficial cervical artery was the most difficult artery to visualize (30). In the present case, to reduce hematoma, seroma, and “dog ear” formation, a Penrose drain pexy was applied. Confirming the vessels except at the base of the flap was difficult. Therefore, considering the damage to major vessels, the vessel was pexied. Additional imaging studies, such as preoperative angiography, would have been helpful in confirming the vascular anatomy (24).

Assessment of flaps viability was made 10 days, 4 and 8 weeks postoperative and was based on skin color and hair growth (31). Subjective evaluation of wound healing is based on physical observations, such as color, odor, and presence of exudates in the recipient wound bed and skin flap (32). In this case, flap viability was evaluated 7 days, 14 days, and 10 months after surgery, and the evaluation was based on skin color, odor, exudate, hair growth, and hair direction.

Our objective was to evaluate the superficial cervical axial pattern flap for opening of the ear canal and the flap’s area, and to evaluate flap elevation and flap rotation. The superficial cervical axial pattern flap was used extensively for head and neck reconstruction (33, 34). The flap can be captured reliably from a single pedicle both left and right (6). These experiments of the superficial cervical axial pattern skin flap were modified to cover a much larger portion of the palate (34). Our study suggest that the contralateral superficial cervical axial pattern flap did not cover the ear canal and did not lead to circulatory problems. The Ipsilateral superficial cervical axial pattern flap resulted in a limitation of rostral reach of the flap due to loss of pliability for opening the ear canal.

In conclusion, the contralateral superficial cervical axial pattern flap and Penrose drain pexy can be effectively applied to large skin defects of the head and neck, including unilateral auricles, to reduce complications and enhance the survival rate.

## Data Availability

The original contributions presented in the study are included in the article/supplementary material, further inquiries can be directed to the corresponding author.
